# Transumbilical cord access (TUCA) for laparoscopy in infants and children: simple, safe and fast

**DOI:** 10.1007/s00595-015-1191-6

**Published:** 2015-06-02

**Authors:** Ralf-Bodo Tröbs, M. Reza Vahdad, Grigore Cernaianu

**Affiliations:** Department of Pediatric Surgery, Catholic Foundation Marienhospital Herne, Ruhr-University of Bochum, Widumer Str. 8, 44627 Herne, Germany; Department of Pediatric and Adolescent Surgery, Klinikum Der Stadt Köln, Amsterdamer Str. 59, 50735 Cologne, Germany; Department of Pediatric Surgery, Universitätsklinikum Schleswig–Holstein, Ratzeburger Allee 160, 23538 Lübeck, Germany

**Keywords:** Open laparoscopy, Pediatric surgical training, Umbilical cord, Cyanoacrylate

## Abstract

**Purpose:**

We herein report a case series evaluating the safety and complication rate of transumbilical cord access (TUCA) for pediatric laparoscopic surgery.

**Methods:**

Data were collected for 556 infants and children. Access into the abdominal cavity was gained via a transverse infraumbilical stab incision passing the fibrotic umbilical cord remnant. Ninety-two infants underwent laparoscopic pyloromyotomy (LPM), 159 female infants underwent herniorrhaphy (LHR) and 309 infants underwent appendectomy (LAP). Of the total operations, 70 % were performed by board-certified surgeons and 30 % were performed by non-board-certified surgeons. The median time of follow-up was 24 months.

**Results:**

No cases of acute severe bleeding or organ laceration were noted. TUCA-related complications were observed in nine patients (1.6 %). Omphalitis and persistent wound secretion were detected in eight children and foreign bodies consisting of cyanoacrylate were removed from three of these patients. Meanwhile, umbilical pain leading to surgical revision was observed in one child, and eight umbilical hernias were repaired during the TUCA procedures. No signs of postoperative incisional hernia were recorded.

**Conclusions:**

TUCA is a safe and comfortable access method for pediatric laparoscopic surgery in various age groups. This method is easy to learn and can be quickly and safely performed in the vast majority of children.

## Introduction

Safe entry into the abdominal cavity and the creation of capnoperitoneum via CO_2_-insufflation are the basic steps that allow for laparoscopic surgery. This maneuver moves the gut and underlying organs away from the abdominal wall, and allows for the safe insertion of additional ports and instruments. Access to the peritoneal space can be gained either by blind percutaneous puncture with a spring-loaded Veress needle (according Janos Veress, 1938) or by using an open approach [1, 2]. For many surgeons, open infraumbilical access based on the method of Hasson is considered the standard approach in adults, and this approach is also often applied in children [3–5].

Potential primary and locoregional complications that may occur during abdominal wall puncture and insufflation of gas include bleeding from the anterior abdominal wall vessels, bowel perforation, visceral vessel bleeding and/or major retroperitoneal vascular injury [6]. Secondary complications include wound infection, incisional hernia development, bowel adhesion, and hypertrophic scar formation.

The aim of the present study was to evaluate our experiences with transumbilical cord access (TUCA) in three different procedures and age groups to analyze the method’s feasibility, safety and complication profile. In addition, we investigated the value of TUCA for pediatric surgical training.

## Methods

In a prospective evaluation, we identified 1,083 laparoscopic procedures using the TUCA approach performed during a 5-year period between 2007 and 2012. For a further investigation, we extracted data from all cases of infants and children who underwent laparoscopic pyloromyotomy (LPM), laparoscopic herniorrhaphy (LHR) or laparoscopic appendectomy (LAP). We collected data on age, gender, complications and follow-up, and furthermore, evaluated the surgeons’ qualifications. Using the International Classification of Procedures in Medicine (ICPM) codes, data were extracted from the hospital documentation system. For follow-up, patient records were examined. The Ethics Committee of the Ruhr-University of Bochum approved this study [Registr. No. 4601-13 (LPM), 4600-13 (LHR) and 4599-13 (LAP)].

The length of time required to establish pneumoperitoneum was estimated in a subset of patients. This time included the initial incision, trocar insertion and confirmation of proper intra-abdominal positioning with the introduced camera. The data for the age at surgery, body weight and length were extracted from the patients’ records. The body mass index (BMI) was estimated according the equation BMI = body mass (kg)/square of body high (m)^2^.

### TUCA procedure

**Fig. 1:**
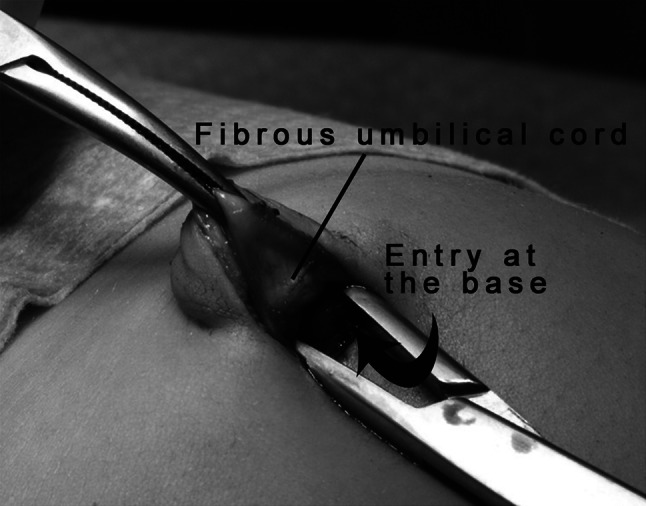
The approach: a clamp is placed at the umbilical cord pillar, and the deep incision is spread using curved forceps

**Fig. 2:**
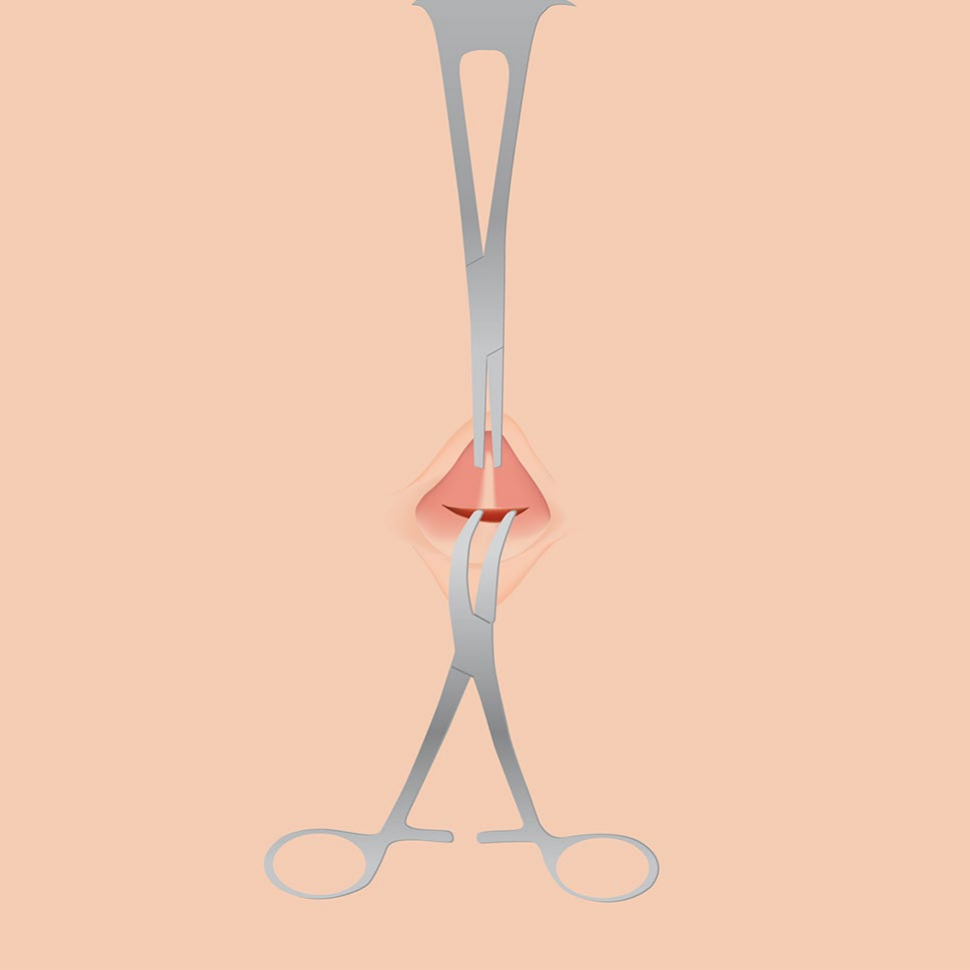
Traction with a clamp and transverse incision at the border between the linea alba and the fibrous umbilical cord. Schematic drawing

Meticulous cleansing and disinfection of the umbilicus were first performed. A stab or “smile” incision was then created at the lower rim of the umbilicus, and the fibrotic remnant of the umbilical cord was freed from the surrounding tissue on the lateral sides. Access into the abdominal cavity was gained via a horizontal incision at the base of the umbilical cord (Fig. 1). A curved Kelly clamp was then placed subcutaneously for traction to facilitate access into the peritoneal cavity using blunt, curved forceps. In children or adolescents with pronounced adiposity, it is beneficial to place a second Kelly clamp at the linea alba below the point of deep incision (Fig. 2).

Blunt spreading of the preperitoneal space and penetration of the peritoneum was used to avoid unintended dissection of the embryological remnants and abdominal contents. The trocar was then placed via an introducer, while purse string sutures were only used in selected cases. The creation of capnoperitoneum was initiated after inserting the camera and checking the intraperitoneal position to avoid subcutaneous emphysema and extraperitoneal insufflation. After the operation, the umbilical wound was closed in layers with single stitches used for the fascia and subcutaneous tissue. Finally, the skin was closed with intracutaneous sutures or cyanoacrylate skin adhesive.

For laparo-endoscopic single-site-surgery (LESS), we used the Triport^**®**^ (Olympus, Hamburg, Germany). The introduction of this trocar system requires transverse enlargement of both the umbilical skin incision and deep fascial entry to approximately 15 mm.

### Statistics

The data are expressed as the median, arithmetic mean ± standard deviation (SD) and [minimum/maximum]. For the statistical analyses, we used a linear regression analysis and Pearson’s correlation coefficient. The significance level for the correlation coefficient was tested for (*n* − 2) degrees of freedom.

## Results

Valid data were extracted from 556 patients with a median follow-up of 24 months (27 ± 14). The age at surgery ranged from 10 days to 17 years. Data for 240 male and 316 female children were included. The body weight (BW) ranged between 2.7 and 106 kg. TUCA was performed in all patients, and no conversion was required.

The number of patients, gender, age, follow-up, and complication rate are presented in Table 1.

**Table 1:** Patient characteristics, follow-up time and complication rate

Procedure	LPM	LHR	LAP
Number of pts.	92 (16.5 %)	155 (28 %)	309 (55.5 %)
Gender (male:female)	78:14	0:155	162:147
Median age	38 days	12 months	11 years
Follow-up	24 months		
Complications	4.3 %	0.6 %	1.3 %
Non-board-certified surgeons	21 %	21 %	37 %

### Laparoscopic pyloromyotomy

This cohort consisted of 78 male and 14 female infants operated on at a median age of 38 days (42 ± 18; range 10–109). LPM was performed with the conventional 3-trocar technique (3LPM; *n* = 71) or LESS (*n* = 21). Complications were observed in four cases (4.3 %).

### Laparoscopic herniorrhaphy

This group exclusively comprised 155 girls with a median age of 12 months (39 ± 50; range 0–207). We applied laparoscopic extraperitoneal repair according to the method of Endo et al. [7] (the conventional open technique was used in boys). The oldest patient in the LHR group was a 17-year-old adolescent girl. One complication (0.6 %) was identified in the LHR group.

### Laparoscopic appendectomy

In this group, 309 children (162 boys and 147 girls) were included. The median age at surgery was 11 years (10 ± 3; range 1–17). Four complications (1.3 %) were observed.

### Umbilical hernia (UH)

In eight cases, LHR patients presented with UH. These children had a median age of 6 months (1–48). Among these cases, the preformed fascial defect was used for trocar insertion, and hernia repair was conducted during wound closure. No recurrence was observed, and no umbilical hernias were encountered in the LPM and LAP groups.

### Types of complications

We experienced no relevant events during trocar insertion requiring additional surgical procedures. No cases of mild emphysema, bleeding or superficial tears of the omentum or bowel surface without further consequences were detected in this retrospective study.

The overall complication rate was 1.6 % (9 of 556). Complications occurred after LHR (one child), LPM and LAP (four children each). During follow-up, five children suffered from umbilical infections, and iatrogenic subcutaneous deposits of the skin adhesive (Dermabond^®^, Ethicon, Norderstedt, Germany) were noted in three infants. In one additional case, umbilical pain led to surgical revision of the umbilicus with removal of the subcutaneous suture material. No postoperative incisional umbilical hernias occurred.

Non-board-certified surgeons performed 30 % of the operations under supervision (167 operations: 19 LPM; 33 LHR; 115 LAP), whereas board-certified surgeons performed 70 % of the operations.

### Time to beginning of insufflation

**Fig. 3:**
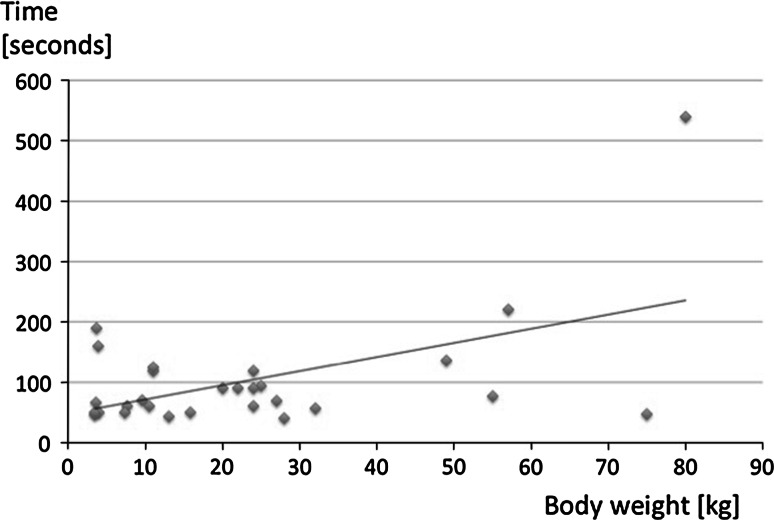
Regression analysis between first trocar insertion time and body weight

In a supplemental group of 28 children with a median body weight of 18 kg (23 ± 22; range 3.4–80), median age of 4.6 years. (5.8 ± 5.4; range 0.05–15.7) and median BMI of 15.4 kg/m^2^ (16.7 ± 4.6; range 11.2–28.6) we estimated the time from the beginning of the incision to the beginning of insufflation. The median time to the start of the creation of pneumoperitoneum was 70 s (102 ± 97; range 46–220). We experienced one case with an extreme time of 540 s in an adipose girl with a body weight of 80 kg (BMI 22 kg/m^2^) and an unfavorable umbilical anatomy. A good correlation was observed between the body mass (BM) and time to gain access (*T*) according to the equation *T* = 47 + 2.3 BM (*r* = 0.52; *p* = 0.01) (Fig. 3). Neither the age at surgery nor BMI correlated with the time required for the first trocar insertion.

## Discussion

The type of access chosen for pediatric laparoscopic procedures is a matter of personal experience and preference. The procedure should be safe, simple and rapid, and both open and closed primary trocar insertion are related to the potential danger of perforating injuries [6]. In a Cochrane Database study, the authors concluded that there “appears to be no evidence of benefit in terms of safety of one technique over another” [8].

Closed laparoscopy can be used safely, and many pediatric surgeons feel comfortable with open access [2, 9, 10]. Surgeon experience and case volume appear to play a major role in preventing complications with closed Veress needle or open access [11]. In a survey of the urologic members of the American Academy of Pediatrics, 45 % exclusively used Veress needle access [12]. However, the survey revealed a higher significant complication rate for the closed technique. In another series, closed access with a Veress needle and blind trocar insertion were the only factors associated with serious complications [13]. Meanwhile, in a Japanese survey, 96.6 % of surgeons preferred minimal open laparotomy for the first trocar insertion in adults [14].

Different techniques for open umbilical access have been described. TUCA uses the inner umbilical ring as a natural, preformed entry into the abdomen. This entry is approached via a short infraumbilical “smile” incision along a natural skin crease. The passage of the trocar is virtually oblique from the infraumbilical skin crease through the inner ring. This approach is advantageous because it provides entry into the abdomen through a former embryological entry, which offers fast and direct access into the abdomen. The resulting scar is nearly invisible in most cases.

In contrast to the findings of previous reports, in which the umbilical cicatrix pillar was incised vertically, we performed a deep transverse incision at the border between the linea alba and the umbilical cord remnant (Fig. 2) [15, 16]. This deep incision line is anatomically preformed in infants and children; it allows for good identification and preservation of embryological duct remnants and facilitates the closure of physiological umbilical hernias in young patients.

Anatomically, the umbilicus is subdivided into three layers: (1) umbilical skin; (2) Wharton’s jelly, which transforms into a fibro-aponeurotic cord during the first year; and (3) the umbilical fascia and internal peritoneal surface [17]. From birth to adulthood, involution of the umbilical ducts occurs. The fibrous remnant of the umbilical cord is not well described in the anatomical literature. Lal et al. [17] introduced the term “umbilical pillar or tube” to describe this structure. However, in a later paper focused on adults, the umbilical duct remnants were not taken into account. In the first months after birth, the urachus, both umbilical arteries and umbilical vein transform into fibrous cords. However, the embryological ducts are vital and should be preserved or ligated in neonates and infants. The relevant rate of “physiological” umbilical hernias in infants allows for easy access. Even in a high proportion of older individuals, the umbilical fascia is absent. The increasing strength of the umbilical fascia and the attachment of the falciform ligament behind the umbilical ring may sustain the mechanical properties of the umbilicus [18, 19]. Therefore, obtaining passage through the internal umbilical ring in older children, adolescents and obese patients may be more difficult [16].

In a subset of our patients, small umbilical hernia sacs were present at laparoscopy and temporarily used for trocar insertion. After the procedure, meticulous closure of the fascia using simple single layer stitches was sufficient; no postoperative umbilical hernias were observed in our series. Furthermore, we always avoided passage directly through the umbilical fossa, as previously described (“umbilical center insertion method”), because we feel that sufficient closure of the fascia may be more difficult with this technique [4, 20, 21].

Various different objective factors may influence the time to gain access, including multiple anatomical layers in the linea alba [3], obesity and the body mass index [16], the diameter of the trocar [21] and previous abdominal surgery.

Although time is not a primary criterion when the objective of the method is simplicity [16], we estimated the time from the beginning of the infraumbilical incision and insufflation in a subset of patients. For the described technique, we estimated a median trocar insertion time of 70 s. In addition, we observed a good correlation with body weight (Fig. 3). In adults, the blind Veress technique required between 3.6 and 5 min for abdominal cavity access [22–24]. Compared with that noted in adults, most pediatric series have reported a shorter time to insertion of the trocar, independent from the applied technique. Our times are comparable with pediatric data collected in the literature (Table 2).

**Table 2:** Trocar insertion, procedural time and complications, overview of selected series

References	Access	Site	Number of patients/procedures	Age	Time to gain access	Complications
[3]	Open, Hasson cannula	Linea alba	14	No data	Not mentioned	No
[19]	Open	Transumbilical, lower half of the umbilicus	>100	5 years (10 days to 16 years)	1 min	0.57 % wound infection
[16]	Open	Supra- or infraumbilically, linea alba	525	No data	4 min (2–12)	?
[9]	Veress needle	Umbilicus	257	4 months to 19 years	<2 min	7 % preperitoneal insufflation
[15]	Open	Supra- or infraumbilically	100	7–84 years	1.55 (0.5–10)	No intra-abdominal injuries
[20]	Open	Umbilical center	431	3.8 years (17 days to 21 years)	1–2 min	No
[8]	Optical access trocar	Umbilicus	14	4.5 ± 2.9 years	1.1 ± 0.8 min	No
Reported series	Open	Umbilicus	556	10 days to 17 years	1.1 min (1.8 ± 1.7 min)	1.6 % omphalitis, foreign body

In the current series, we observed no complications specifically related to the access procedure and no cases of laceration of the embryological ducts, bowel, liver or vessels. If in doubt, the surgeon should coagulate or ligate the embryological umbilical remnant before dissection. Some authors recommend a lateral approach to the umbilical ring to avoid laceration of the urachus remnant [4]. In cases of periumbilical dermatitis, umbilical secretions or subumbilical cysts, the use of TUCA may be contraindicated. Excluding preperitoneal insufflation, our complication rate of 1.6 % was somewhat higher than that reported in other series. In a North American survey, a complication rate of 1.18 % was reported [12]. However, available investigations did not focus on umbilical infections or stone formation. Furthermore, the role of umbilical bacterial flora in the onset of surgical site infections in infants and children has not yet been studied.

Therefore, in newborn infants with incomplete involution of the umbilical cord stump, the need for perioperative antibiotic prophylaxis (AP) must be taken into account (level of evidence IV). Generally, there is no existing evidence supporting the use of AP for hernia repair or pyloromyotomy.

Because antibiotics have been demonstrated to be effective in preventing postoperative complications after appendectomy, all patients in this subgroup received antibiotic prophylaxis [25]. Data confirm the hypothesis that the grade of appendiceal inflammation correlates with the rate of surgical site infection [26]. In this series, we did not analyze the severity of appendiceal inflammation.

The surgeon should be aware that the use of non-absorbable cyanoacrylate skin adhesive for closure of the trocar wound along the umbilical skin crease carries a risk of postoperative subcutaneous sealant stone formation, foreign body reactions and infection. Hence, precise approximation of the skin edges for 10 s is required to prevent the formation of subcutaneous sealant deposits. In suspicious cases, surgical revision with removal of the sealant is curative.

In recent years, there has been a clear tendency to use umbilical single-site laparoscopic techniques in infants, children, adolescents and adults [27–29]. In the present series, we applied laparo-endoscopic single-site surgery in subsets of infants and children treated with appendectomy and pyloromyotomy. Laparoscopic herniorrhaphy was performed using a single-site technique employing a needle instrument for the insertion of inguinal purse string sutures [7].

Laparoscopic techniques represent an important component of pediatric surgical training. In this series, nearly one-third of the operations were primarily performed by non-board-certified surgeons under the assistance of a certified surgeon. Our experience indicates that the TUCA technique is both easy to teach and simple to learn.

In conclusion, the transumbilical cord access technique is a simple, safe and rapid access method for primary trocar insertion, and the rate of procedure-related complications associated with TUCA is minimal.

## References

[1] Levitt MA, Tantoco JG, Najmaldin A (2005). Instruments for laparoscopy and thoracoscopy access. Operative endoscopy and endoscopic surgery in infants and children.

[2] Schier F, Turial S (2013). Laparoscopy in children.

[3] Hasson HM (1971). A modified instrument and method for laparoscopy. Am J Obstet Gynecol.

[4] Schleef J, Bax MA, Georgeson KE, Rotheberg SS, Valla JS, Yeung CK (2008). Complications of endoscopic surgery in infants and children. Endoscopic surgery in infants and children.

[5] Poppas DP, Bleustein CB, Peters CA (1999). Box stitch modification of Hasson technique for pediatric laparoscopy. J Endourol.

[6] Schäfer M, Lauper M, Krähenbühl L (2001). Trocar and Veress needle injuries during laparoscopy. Surg Endosc.

[7] Endo M, Watanabe T, Nakano M, Yoshida F, Ukiyama E (2009). Laparoscopic completely extraperitoneal repair of inguinal hernia in children: a single-institute experience with 1,257 repairs compared with cut-down herniorrhaphy. Surg Endosc.

[8] Ahmad G, Duffy JM, Philips K, Watson A. Laparoscopic entry techniques. Cochrane Database Syst Rev. 2008; 2:CD006583. 10.1002/14651858.CD006583.pub2 18425957

[9] Silay MS, Tepeler A, Sancaktutar AA, Kilincaslan H, Altay B, Erdem MR (2013). The all-seeing needle instead of the Veress needle in pediatric urologic laparoscopy. J Endourol.

[10] Yanke BV, Horowitz M (2007). Safety of the Veress needle in pediatric laparoscopy. J Endourol.

[11] Passerotti CC, Nguyen HT, Retik AB, Peters CA (2008). Patterns and predictors of laparoscopic complications in pediatric urology: the role of ongoing surgical volume and access techniques. J Urol.

[12] Peters CA (1996). Complications in pediatric urological laparoscopy: results of a survey. J Urol.

[13] Mayol J, Garcia-Aguilar J, Ortiz-Oshiro E, De-Diego Carmona JA, Fernandez-Represa JA (1997). Risks of the minimal access approach for laparoscopic surgery: multivariate analysis of morbidity related to umbilical trocar insertion. World J Surg.

[14] Hashizume M, Sugimachi K (1997). Needle and trocar injury during laparoscopic surgery in Japan. Surg Endosc.

[15] Scott-Conner CEH, Dawson DL, Scott-Conner CEH, Dawson DL (2009). Laparoscopy: principles of access and exposure. Operative anatomy.

[16] Moberg AC, Petersson U, Montgomery A (2007). An open access technique to create pneumoperitoneum in laparoscopic surgery. Scand J Surg..

[17] Lal P, Sharma R, Chander R, Ramteke VK (2002). A technique for open trocar placement in laparoscopic surgery using the umbilical cicatrix tube. Surg Endosc.

[18] Fathi AH, Soltanian H, Saber AA (2012). Surgical anatomy and morphologic variations of umbilical structures. Am Surg.

[19] Oh CS, Won HS, Kwon CHD, Chung IH (2008). Morphologic variations of the umbilical ring, umbilical ligaments and ligamentum teres hepatis. Yonsai Med J..

[20] Esposito C (1997). Transumbilical open laparoscopy: a simple method of avoiding complications in pediatric surgery. Pediatr Surg Int.

[21] Nakaoka T, Uemura S, Yoshida T, Tanimoto T, Shiokawa C, Harumoto K. Umbilical center insertion method for initial trocar placement in pediatric laparoscopic surgery. Osaka City Med J. 2010; 56:21–6 **(abstract)**. 21466126

[22] Bonjer HJ, Hazebroek EJ, Kazemier G, Giuffrida MC, Meijer WS, Lange JF (1997). Open versus closed establishment of pneumoperitoneum in laparoscopic surgery. Br J Surg.

[23] Borgatta L, Gruss L, Barad D, Kaali SG (1990). Direct trocar insertion vs. Veress needle use for laparoscopic surgery: a French survey of 103 852 operations. J Reprod Med.

[24] Cogliandodlo A, Manganaro T, Saitta FP, Micali B. Blind vs. open approach to laparoscopic cholecystectomy: a randomized study. Surg Laparoscop Endosc. 1998;8:853–5. 9799143

[25] Andersen BR, Kallehave FL, Andersen HK. Antibiotics versus placebo for prevention of postoperative infection after appendectomy. Cochrane Database Syst Rev. 2001;20:CD001439. doi: 10.1002/14651858.CD001439. 10.1002/14651858.CD001439 11686991

[26] Sohn M, Hoffmann M, Hochrein A, Buhr HJ, Lehmann KS. Laparoscopic appendectomy is safe: influence of appendectomy technique on surgical-site infections and intra-abdominal abscesses. Surg Laparosc Endosc Percut Tech. 2014 **(epub ahead of print)**. 10.1097/SLE.0000000000000115 25462984

[27] Li B, Chen WB, Wang SQ, Wang YB (2015). Single-site umbilical laparoscopic pyloromyotomy in neonates less than 21 days old. Surg Today.

[28] Kimura T, Yamauchi K, Ihara Y, Sawai T, Kosumi T, Yonekura T (2012). Single-site laparoscopic herniorrhaphy using needle instruments for inguinal hernias in children: a novel technique. Surg Today.

[29] Yamazaki M, Yasuda H, Koda K. Single-incision laparoscopic cholecystectomy: a systematic review of methodology and outcomes. Surg Today 2014 Mai 22. doi:10.1007/s00595-014-0908-2. 10.1007/s00595-014-0908-2 24845737

